# Dr. Lucien Damainville

**Published:** 1892-01

**Authors:** 


### Obituary.

---

Dr. Lucien Damainville died in New York, on Tuesday, December 15, 1891, aged fifty-two years. Dr. Damainville was a former resident of Buffalo, and it will be remembered by old Buffalonians, that his father was one of the prominent Frenchmen early identified with the history of the city. Lucien himself was a student of the late Dr. Frank Hastings Hamilton when he was professor of surgery in the University of Buffalo, and was a classmate and companion of the writer during the latter part of the fifties.

When Dr. Hamilton went to New York, Dr. Damainville also carried his fortunes hitherward, where he was more or less associated with his chief for many years. They entered the military service substantially together, Dr. Hamilton as surgeon of the thirty-first regiment, New York volunteers, and Dr. Damainville, as assistant-surgeon in the same regiment. The regiment was commanded by Colonel — now judge — Calvin E. Pratt, of Brooklyn, who presided recently at the New England dinner given at the Brooklyn Academy of Music. Judge Pratt and Dr. Hamilton, as well as Dr. Damainville, were always warm personal friends, hence this allusion to their relationship.

After Dr. Hamilton's promotion to be surgeon of United States volunteers, Dr. Damainville succeeded him as surgeon of the thirty-first, in which office he continued until the expiration of the term of enlistment of the regiment. After the war, Dr. Damainville returned to New York, and has continued in the practice of his profession in that city until his death, serving for a number of years as Surgeon of Police. He visited Buffalo in 1883, in company with Dr. Hamilton, which was the latter's last visit to his old home, and it is the impression of the writer that Dr. Damainville himself has not visited Buffalo since that time.

Dr. Damainville was a man of positive convictions, able in his profession, and studious in his habits. We mourn his loss as one of his friends as well as his colleague in the profession that he loved so well.

